# Three‐dimensional dose prediction based on two‐dimensional verification measurements for IMRT

**DOI:** 10.1120/jacmp.v15i5.4874

**Published:** 2014-09-08

**Authors:** Iori Sumida, Hajime Yamaguchi, Hisao Kizaki, Keiko Aboshi, Yuji Yamada, Yasuo Yoshioka, Kazuhiko Ogawa

**Affiliations:** ^1^ Department of Radiation Oncology Osaka University Graduate School of Medicine Osaka Japan; ^2^ Department of Radiation Oncology NTT West Osaka Hospital Osaka Japan

**Keywords:** IMRT, dose prediction, step and shoot, dosimetry, QA

## Abstract

Dose verifications for intensity‐modulated radiation therapy (IMRT) are generally performed once before treatment. A 39‐fraction treatment course for prostate cancer delivers a dose prescription of 78 Gy in eight weeks. Any changes in multileaf collimator leaf position over the treatment course may affect the dosimetry. To evaluate the magnitude of deviations from the predicted dose over an entire treatment course with MLC leaf calibrations performed every two weeks, we tracked weekly changes in relative dose error distributions measured with two‐dimensional (2D) beam‐by‐beam analysis. We compared the dosimetric results from 20 consecutive patient‐specific IMRT quality assurance (QA) tests using beam‐by‐beam analysis and a 2D diode detector array to the dose plans calculated by the treatment planning system (TPS). We added back the resulting relative dose error measured weekly into the original dose grid for each beam. To validate the prediction method, the predicted doses and dose distributions were compared to the measurements using an ionization chamber and film. The predicted doses were in good agreement, within 2% of the measured doses, and the predicted dose distributions also presented good agreement with the measured distributions. Dose verification results measured once as a pretreatment QA test were not completely stable, as results of weekly beam‐by‐beam analysis showed some variation. Because dosimetric errors throughout the treatment course were averaged, the overall dosimetric impact to patients was small.

PACS numbers: 87.55.D‐, 87.55.dk, 87.55.km, 87.55.Qr

PACS numbers: 87.55.D-, 87.55.dk, 87.55.km, 87.55.Qr

## I. INTRODUCTION

Intensity-modulated radiation therapy (IMRT) is an important method of maximizing target dose while minimizing the dose to the surrounding normal tissues. Treatment fields are highly complex, however, necessitating quality assurance (QA) to verify both the accuracy of the treatment planning system (TPS) and the performance of the beam delivery system. Pretreatment QA measures per-beam and/or composite dose distributions, as well as absolute dose measurements. The accuracy of beam delivery by the segmental multileaf collimator (MLC) method is particularly dependent on leaf stop position and in the dynamic MLC method on leaf motion speed precision. Deviation of leaves from the expected stop positions raises the potential for substantial dose error.[(1)] Hence, MLC leaf position QA should be performed at appropriate intervals, as recommended by the AAPM Task Group 142.[(2)] Our department performs a weekly Garden Fence test, and also the Picket Fence test (also called the "nongap

[a] Corresponding author: Iori Sumida, Department of Radiation Oncology, Osaka University Graduate School of Medicine, 2-2 Yamada-oka, Suita, Osaka, 565-0871 Japan; phone: 81(6) 6879 3482; fax: 81(6) 6879 3489; email: sumida@radonc.med.osaka-u.ac.jp test"),[3,4] a similar test with the leaves closed, to verify MLC leaf stop position and to measure relative dose perturbation at MLC leaf abutments using step-and-shoot leaf motion. Results have shown that MLC leaf position gradually changes as much as 1 mm from baseline over the course of one month, and that relative dose intensity at the MLC leaf abutments consequently increases or decreases compared to that specified by the TPS.[3] The MLC used in this study is controlled by a potentiometer and an encoder to recognize leaf positions. The potentiometer has an absolute current value and the encoder has a relative value. The treatment machine is also equipped with a feature that stops the MLC leaves moving toward the isocenter once the field size changes according to the manufacturer's hardware control system. If the treatment machine is turned off at the end of the workday and turned on again the following morning, the encoder is initialized. Following initialization, while the absolute current value for the potentiometer of each MLC leaf does not change, the MLC leaf position is moved toward the closing field via integration of the on/off switching procedure of the system. These findings suggest that more frequent MLC leaf calibration may be necessary to deliver an accurate dose to a patient for a whole treatment course.

Per-beam QA using a two-dimensional (2D) diode detector array is usually performed at a gantry angle of 0° in the coronal plane (coronal plane QA). However, Nelms et al.[5] reported that planar IMRT QA passing rates are not predictive of clinically relevant patient dose errors, and Kruse[6] found that gamma analysis of single field measurements is insensitive to important dosimetric inaccuracies in the overall plan. At present, dose errors taken from the 2D detectors are fed back into the TPS in three dimensions (3D), and then the predicted dose errors are added to the original calculated dose.[7,8,9] As a rule, a single IMRT dose verification before treatment is an excellent indication of delivery over the whole treatment course, and multiple verifications are not considered necessary.[10] However, additional dose errors might result from MLC leaf positional deviations over a long treatment period. This leads to the possibility that dose errors in 3D might change after factoring in the clinically relevant dose-volume indices.

We investigated the reliability of dose delivery in 3D using the 2D dose errors calculated from per-beam QA, and predicted the clinically relevant dose volume indices based on these dose errors throughout the radiation treatment course. We focused on prostate cancer IMRT with 78 Gy/39 fractions. We performed weekly MLC QA using an electronic portal imaging device (EPID) and per-beam QA using 2D diode detector arrays, and performed MLC leaf calibration every two weeks.

## II. MATERIALS AND METHODS

### A. Treatment planning

Treatment with five fields was done using a 10 MV linear accelerator (ONCOR Impression Plus; Siemens Medical Systems, Concord, CA). A leaf width of 10 mm was used for step-and-shoot delivery. A total of 20 prostate cancer patients treated by IMRT were included in the study. After a physician delineated the contouring of the CTV with the prostate as a target and the bladder and rectum as critical organs, a medical physicist created the plan with the TPS (XiO, ELEKTA, Stockholm, Sweden). Dose calculation was performed with a grid size of 2 mm. A mean dose of 78 Gy to the prostate planning target volume (PTV) over 39 fractions was prescribed in all patients. The mean equivalent side of each segment for all patients was 4.49 ± 0.37 cm (mean ± 1 SD).

### B. Data export

For dose distribution analysis using a 2D detector diode array (MapCHECK; Sun Nuclear Corporation, Melbourne FL), one fraction of the treatment dose for each patient was calculated in a $30 \times 30 \times 30$ cm$^3$ thick Solid Water phantom at a gantry angle of 0° instead of the actual treatment gantry angle, assuming that there was no drift of the MLC leaf stop positions with different angles. Source-to-detector distance was 100 cm and depth was 10 cm. After the dose calculations were performed with a 1 mm grid, the dose distribution in the coronal plane for each beam at a depth of 10 cm was exported in the text format used by the TPS. The Digital Imaging and Communications in Medicine-Radiation Therapy (DICOM RT) plan, DICOM RT structure set, and DICOM RT dose for each beam were exported for the evaluation of dose-volume indices in the treatment plan for each patient.

## C. Creation of error map

We developed an in-house software application using Delphi2007 (Borland Software Corporation, Austin TX) to create a 2D error map of calculated versus measured dose distribution, and to adapt the 2D error map to the 3D DICOM RT dose grid. The 2D diode detector array was placed on the treatment couch with a 10 cm water-equivalent total phantom thickness and a source–detector distance of 100 cm. The reproducibility of absolute dose for the 2D diode detector array before and after beam-by-beam measurement for each patient was checked with a $10 \times 10$ cm$^2$ field at a depth of 10 cm water equivalent. Variations did not exceed 0.3% during the study. After the measurements for each patient with the 2D diode detector array at a gantry angle of 0°, the measured dose distributions were exported by MapCHECK in the text file format. Those files were imported into our in-house software and were compared with those calculated by the TPS. The spatial resolution of measurements using the 2D diode detector array was 5 mm; therefore, the calculated dose at the same position for the 2D diode detector array was selected. The relative local dose error map for dose differences between measurements and calculations was created using the following equation:

$$Dose\ error = \frac{Measured\ dose\ (cGy) - Calculated\ dose\ (cGy)}{Calculated\ dose\ (cGy)} \quad (1)$$

According to the equation, the error map for each beam was exported with a grid size of 5 mm.

We used the gamma analysis method to evaluate dose distribution.(11,12) Analysis was limited to doses greater than 10% of the maximum dose on the TPS. The absolute dose and distance-to-agreement tolerances were 3% and 3 mm, respectively. The degree of agreement between the 2D diode detector array and the TPS calculation was characterized using the passing rate of diode detectors failing to have gamma < 1. The passing rates for all beams were over 95%, within the tolerance level used in our department to start radiation treatment.

## D. Adaptation of error map to error-free dose grid

Because measurements with the 2D diode detector array were performed at a 0° gantry angle, the error-free dose grid for each beam (i.e., the DICOM RT dose grid of the original plan) was converted into 3D using the isocenter location [$T_x$, $T_y$, $T_z$] in CT coordinates with units expressed in mm and rotated in the z-axis (head–foot direction) using the planned gantry angle θ, referenced in the DICOM RT plan file before the error map was applied. The following equation was used:

$$\begin{pmatrix} D_x \\ D_y \\ D_z \end{pmatrix} = \begin{pmatrix} cos\theta & -sin\theta & 0 \\ sin\theta & cos\theta & 0 \\ 0 & 0 & 1 \end{pmatrix} \begin{pmatrix} S_x \\ S_y \\ S_z \end{pmatrix} + \begin{pmatrix} T_x \\ T_y \\ T_z \end{pmatrix} \quad (2)$$

where ($D_x$, $D_y$, $D_z$) is the destination location of each dose grid, and ($S_x$, $S_y$, $S_z$) is the source location of each dose grid. To determine the projected location in the isocenter plane where the 2D diode detector array measurement was performed at gantry angle 0° for each destination location, the rate of magnification $R_{mag}$ was calculated using the distance between $D_y$ and $T_y$. The following equation was used:

$$R_{mag} = 1 - (D_y - T_y)/1000 \quad (3)$$

The projected locations on the x- and z-axes of the CT coordinates were then calculated using the following equations:

$$Map_x = R_{mag} \times (D_x - T_x) \quad (4)$$

$$Map_z = R_{mag} \times (D_z - T_z) \quad (5)$$

where $Map_x$ and $Map_z$ were the locations on the lateral and craniocaudal detector planes of the 2D diode detector array, respectively. The relative dose error at the point of $Map_x$ and $Map_z$, $Error_{Mapx\ Mapz}$, was calculated from the 2D linear interpolation of the relative dose error at each detector point in the 2D diode detector array's coordinates. The $Error_{Mapx\ Mapz}$ was applied to the error-free dose at each DICOM RT dose grid coordinate (x, y, z), resulting in $Dose_{errorfree\ x,y,z}$ in three dimensions for each beam, producing $Dose_{with\ error\ map\ x,y,z}$, as illustrated by the following equation:

$$Dose_{with\ error\ map\ x,\ y,\ z} = Dose_{errorfree\ x,y,z} \times (1 + Error_{Mapx,Mapz}) \quad (6)$$

The sums of the error-involved dose grid for each beam were then used to evaluate dose-volume indices. The final dose compositions and calculation of dose-volume indices were performed by the in-house software; the dose calculation was not performed by the in-house software solely to use the dose grid for each beam.

## E. Dose validation

The dose validation for the proposed method was performed with one of the 20 patients. Measurements of absorbed dose and verification for isodose distributions in the axial plane using film were performed. The verification phantom named I'mRT Phantom (IBA Dosimetry, Schwarzenbruck, Germany) was used in both verifications. An ionization chamber (PTW PinPoint 31016 chamber; PTW, Freiburg, Germany) was used to measure the absorbed dose. Three sheets of radiochromic film (GAFCHROMIC EBT2; International Specialty Products, Wayne NJ) were inserted in the plane of -1, 0 (isocenter), and +1 cm in the craniocaudal direction to verify the isodose distributions. The treatment beams were overlaid onto the phantom, then five measurement points, including the isocenter, were chosen in the region of high-dose and low-dose gradients. The locations of the measurements of absorbed dose and isodose distributions are shown in Fig. 1. This plan is referred to as the "original plan." In order to create a plan with dose error, the MLC leaf positions of each beam were manually changed. This plan is referred to as the "modified plan." Both the original plan and the modified plan used the same monitor units for each beam. The per-beam QA for the modified plan using the 2D diode detector array was done at the gantry angle of 0°, a source–detector distance of 100 cm, and depth of 10 cm. To create an error map of each beam, the coronal dose plane in the original plan was used as a reference. The error map of each beam was incorporated into the original dose grid based on our proposed method to create a 3D dose grid with MLC leaf error, thus generating predicted dose grid data with dose error. That process is shown in Fig. 2.

The predicted dose at five points was compared with the measured dose and the modified plan dose of the TPS. The predicted isodose distributions of three axial planes were compared with the isodose distributions from the measured films. To verify isodose distributions, three kinds of dose evaluation were used, namely, relative dose difference, distance to agreement, and percent pass rate of gamma function (3%/3 mm criteria).[11,12]

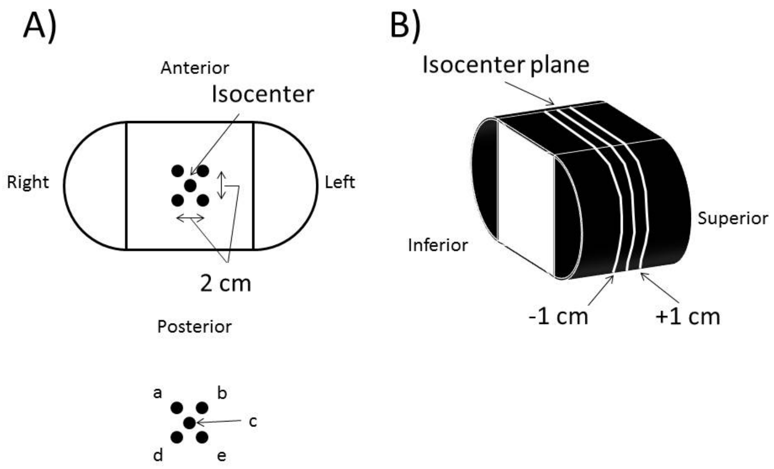

FIG. 1. Measurement locations of absorbed dose and dose distribution are shown. The five black circles show the measurement points (a-e). Four points (a, b, d, e) are 1 cm from the isocenter (c) in the left-to-right direction and anterior-to-posterior direction. Three sheets of film (b) were inserted in three dose planes, namely the isocenter plane and +1 cm and -1 cm to the isocenter plane, in the superior to inferior direction.

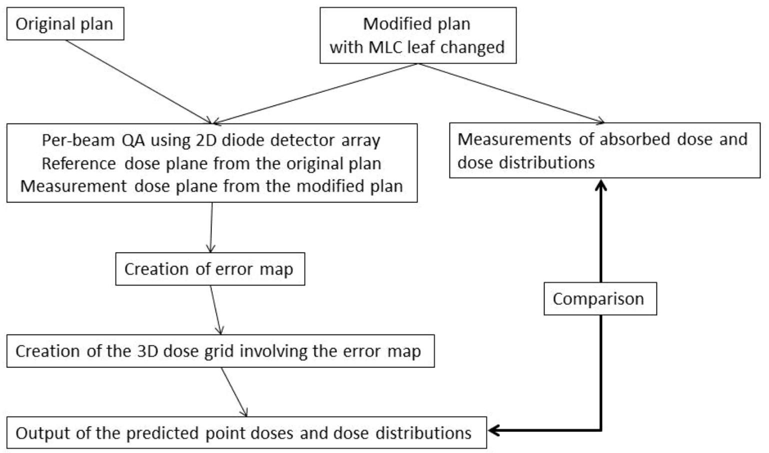

FIG. 2. The dose validation process of our proposed method modification.

## F. QA schedule for dose evaluation

The reproducibility of measurements was evaluated in three of the 20 patients. Measurement of beam-by-beam analysis using the 2D diode detector array was performed ten times on the same day. The same three cases were subjected to weekly dose evaluations throughout the treatment course of 39 fractions over eight weeks. Beam-by-beam analysis using the 2D diode detector array was performed weekly, as well as before and after MLC leaf calibration, which was performed three times every two weeks over eight weeks. All error-involved dose grids and the error-free dose grid were divided by the number of total fractions (39). Therefore each dose grid was based on the prescription dose of 2 Gy.

The QA schedule is summarized in Table 1. We show two hypothetical cases, Case A and Case B. In general the pretreatment QA is performed once before starting the radiation treatment. If the MLC leaf stop position is stable through the whole treatment course, it might be enough to evaluate the dose distributions one time before treatment. However, if the MLC leaf stop position gradually changes during the whole treatment course, as ours did, it might not

Table 1. QA test schedule for the entire treatment course of 39 fractions.

| | | | *Case A* | *Case B* |
|---|---|---|---|---|
| | | | *Number of Fractions* | |
| | | Error-free dose grid | 39 | 39 |
| 1st week | Per-beam QA[a] | Error-involved dose grid 1 | 39 | 5 |
| 2nd week | Per-beam QA | Error-involved dose grid 1 | 39 | 5 |
| 3rd week | Per-beam QA (Pre-calib.)[b] | Error-involved dose grid 3-Pre. | | |
| | Per-beam QA (Post-calib.)[c] | Error-involved dose grid 3-Post. | 39 | 5 |
| 4th week | Per-beam QA | Error-involved dose grid 4 | 39 | 5 |
| 5th week | Per-beam QA (Pre-calib.) | Error-involved dose grid 5-Pre. | | |
| | Per-beam QA (Post-calib.) | Error-involved dose grid 5-Post. | 39 | 5 |
| 6th week | Per-beam QA | Error-involved dose grid 6 | 39 | 5 |
| 7th week | Per-beam QA (Pre-calib.) | Error-involved dose grid 7-Pre. | | |
| | Per-beam QA (Post-calib.) | Error-involved dose grid 7-Post. | 39 | 5 |
| 8th week | Per-beam QA | Error-involved dose grid 8 | 39 | 4 |

[a] Beam-by-beam analysis.
[b] Pre-MLC leaf calibration.
[c] Post-MLC leaf calibration.

be enough to evaluate the dose distribution one time before treatment. Therefore, for Case A it was assumed that the dose errors measured at the beginning of a certain week between the first and eighth week were unchanged over the 39 fractions. The errors were used as a substitute for the single pretreatment QA. That is, each patient could start the treatment between the first and eighth weeks. For Case B, it was assumed that the dose errors measured every week were factored into the cumulative dose over the 39 fractions. Eventually, the final dose included the potential dosimetric change throughout the treatment course, such as the dose perturbation, according to the change in MLC leaf position. Cases A and B were used to judge whether the pretreatment QA was applicable for the patient-specific QA. In terms of the dose evaluations for Case A, each error-involved dose grid of 1, 2, 3-Post (calibration), 4, 5-Post, 6, 7-Post, and 8 was multiplied by 39 fractions and applied over the whole treatment course. For Case B, each error-involved dose grid of 1, 2, 3-Post, 4, 5-Post, 6, and 7-Post was multiplied by 5 fractions, and dose grid 8 was multiplied by 4 fractions. Summing each dose grid then provided a more realistic error-involved dose grid, which took into account the potential dose changes due to MLC leaf positional changes over less than one week.

For dose evaluation with dose-volume indices of the target, $D_{98\%}$ to the CTV and PTV as a minimum dose, $D_{2\%}$ to the CTV and PTV as a maximum dose, mean dose to the CTV and PTV, and $D_{95\%}$ to the PTV were calculated and expressed in Gy. $D_{98\%}$ and $D_{2\%}$ were chosen according to the dose specification protocol of ICRU Report 83.(13) For the organs at risk, the percentage of rectal tissue receiving 65 Gy ($V_{65Gy}$) and the percentage of bladder tissue receiving 40 Gy ($V_{40Gy}$) were calculated. Rectal dose constraints of $V_{65Gy} < 17\%$ and $V_{40Gy} < 35\%$, and bladder dose constraints of $V_{65Gy} < 25\%$ and $V_{40Gy} < 50\%$, based on a previous report,(14) were determined by a physician and used as the planning goal. For both Cases A and B, dose-volume indices were compared between the error-free dose grid as reference and error-involved dose grid. Student's *t*-test was used for comparison. Statistical significance was set at the 5% level.

## III. RESULTS

### A. Dose validation for proposed method

For absolute dose validation for the modified method, the comparisons at the five measurement points between the predicted dose and measurement dose are shown in Table 2. The original planned dose and the modified planned dose were calculated by the TPS. The predicted dose errors against the modified planned dose were 1.16%, -1.09%, -1.52%, -1.09%, and -0.54% at

Table 2. Comparisons of the predicted dose and the measurements dose.

| Point[a] | Original Planned Dose (Gy) | Modified Planned Dose (Gy) | Predicted Dose (Gy) | Measured Dose (Gy) | Predicted Dose Error Against Modified Planned Dose (%) | Predicted Dose Error Against Measured Dose (%) | Measured Dose Error Against Modified Planned Dose (%) |
|---|---|---|---|---|---|---|---|
| a | 2.01 | 1.72 | 1.74 | 1.73 | 1.16 | 0.40 | 0.76 |
| b | 2.02 | 1.84 | 1.82 | 1.86 | -1.09 | -2.20 | 1.14 |
| c | 1.99 | 1.97 | 1.95 | 1.99 | -1.52 | -2.66 | 1.17 |
| d | 1.97 | 1.83 | 1.81 | 1.86 | -1.09 | -2.79 | 1.75 |
| e | 1.99 | 1.85 | 1.84 | 1.90 | -0.54 | -2.95 | 2.49 |

[a] Points a to e are the measurement points in Fig. 1(a).

the measurement points of a, b, c, d, and e, respectively. The predicted dose errors against the measured dose were 0.40%, -2.20%, -2.66%, -2.79%, and -2.95% at the measurement points of a, b, c, d, and e, respectively. The differences between the predicted dose and the measured dose were larger than 2% for the points b to e. However, since the measured dose errors against the modified planned dose were 0.76%, 1.14%, 1.17%, 1.75%, and 2.49% at the measurement points of a, b, c, d, and e, respectively, the predicted dose error against the measured dose could be as small as 1.5%, as the values of the differences were close to the results for the predicted dose error against the modified planned dose.

For dose distribution validation of the proposed method, the isocenter plane as a typical dose distribution is shown in Fig. 3. The predicted dose distribution in Fig. 3(e) was derived

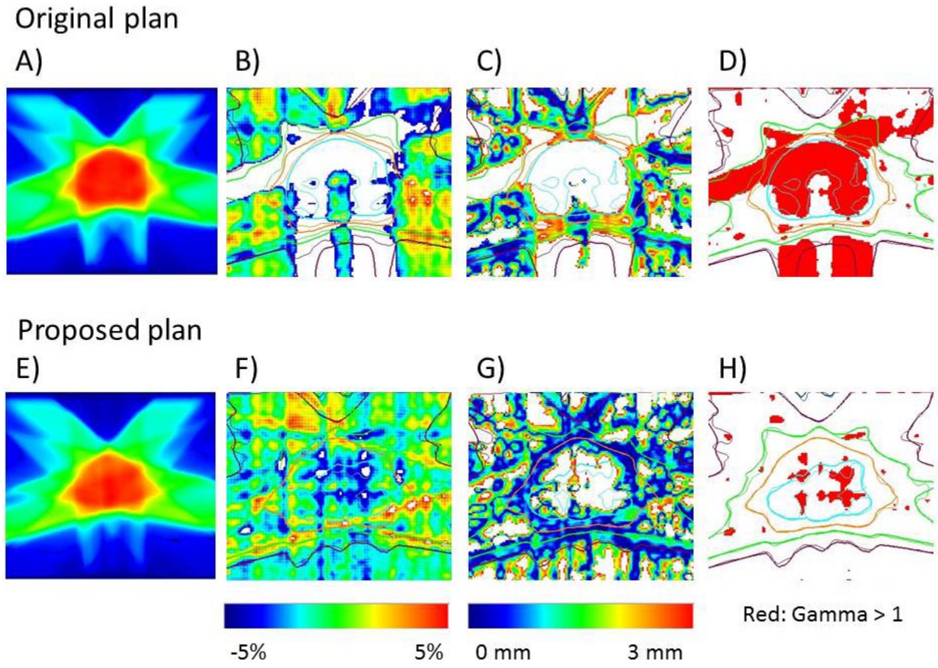

Fig. 3. Comparisons of dose distribution between the original plan, the plan generated by our modified method, and film measurement (all data are at the isocenter plane): (a) represents the original dose distribution; (e) represents the proposed modification plan dose distribution; (b), (c), and (d) are comparisons between (a) and film measurement; (f), (g), and (h) are the comparisons between (e) and film measurement; (b) and (f) are the original and modified dose differences, respectively, with an error range of 5%; (c) and (g) represent the original and modified distance to agreement, respectively, with an error range of 3 mm; (d) and (h) are the original and modified gamma distributions, respectively. Gamma values > 1 are in red.

from the MLC leaf modification that made the leaves close with the same monitor units of the original plan. Therefore, the dose distribution in Fig. 3(e) had a lower dose compared to the original dose distribution in Fig. 3(a). Unfortunately, stripe artifacts on the nonirradiated film, which might lead to an unexpected dose error, were seen in both lateral and vertical directions on the whole measurement area. For the dose difference, the modified plan dose distribution showed better agreement with the film measurement in Fig. 3(f), especially in the region with large dose errors shown in white in Fig. 3(b), which means a relative dose error over 5%. For the distance to agreement, the predicted isodose distributions were well-matched to the measured isodose distributions shown in Fig. 3(g). For the gamma analysis with the tolerance criteria (3%/3 mm), the percent pass rate of the modified plan dose distribution and the film measurement in Fig. 3(h), and that of the original dose distribution and the film measurement in Fig. 3(d), were 86.9% and 67.0%, respectively. The regions of gamma values over 1 in red were mainly due to the inherent film artifacts.

## B. Accuracy measurement

Sequential measurements using the 2D diode detector array were performed ten times for three patients to evaluate measurement reproducibility. Table 3 summarizes the relative dose differences between the mean of ten error-involved dose grid measurements and the error-free dose grid for Cases 1, 2, and 3. For all three cases, the values of $D_{98\%}$, $D_{95\%}$, $D_{2\%}$, and mean dose to the PTV, and $D_{98\%}$, $D_{2\%}$, and mean dose to the CTV calculated using the error-involved dose grid, were statistically different from those calculated with the error-free dose grid ($p < 0.01$, except for Case 2, CTV $D_{98\%}$ and CTV mean dose $p < 0.03$), although the differences among the three cases have not shown a consistent tendency to skew up or down. The values of $V_{65Gy}$ and $V_{40Gy}$ to both the rectum and the bladder, except the $V_{65Gy}$ to the bladder in Case 2, using the error-involved dose grid were also statistically different from those calculated with the error-free dose grid ($p < 0.01$; Case 2, $p < 0.03$), although the difference among the cases, again, did not show a consistent tendency to skew up or down.

TABLE 3. Comparison of error-involved and error-free dose grids for three cases.

| *Volume Indices* | *Case 1 (%)* | *Case 2 (%)* | *Case 3 (%)* | *p-value* |
|---|---|---|---|---|
| PTV $D_{98\%}$ | -1.46±0.09 | -1.66±0.05 | 4.23±0.34 | < 0.01 |
| PTV $D_{95\%}$ | -1.97±0.08 | -1.58±0.06 | -0.24±0.13 | < 0.01 |
| PTV $D_{2\%}$ | 1.47±0.29 | 0.95±0.06 | 2.17±0.18 | < 0.01 |
| PTV mean dose | -0.85±0.05 | 0.24±0.08 | 0.21±0.10 | < 0.01 |
| CTV $D_{98\%}$ | -0.87±0.06 | -0.30±0.27[a] | -5.09±0.25 | < 0.01<br>< 0.03[a] |
| CTV $D_{2\%}$ | -0.58±0.05 | -0.19±0.06 | 2.25±0.21 | < 0.01 |
| CTV mean dose | -0.81±0.05 | -0.10±0.10[a] | -0.60±0.12 | < 0.01<br>< 0.03[a] |
| Rectum $V_{65Gy}$ | -16.99±0.02 | -5.24±0.01 | 42.32±0.37 | < 0.01 |
| Rectum $V_{40Gy}$ | -4.40±0.06 | -1.47±0.07 | 24.23±0.07 | < 0.01 |
| Bladder $V_{65Gy}$ | 14.71±0.03 | 0.37±0.17[a] | 8.69±0.04 | < 0.01 |
| Bladder $V_{40Gy}$ | 10.10±0.01 | -4.63±0.14 | 7.90±0.06 | < 0.01 |

[a] Not significant.

## C. Changes in dose-volume indices over time

For Cases 1, 2, and 3, beam-by-beam analysis using the 2D diode detector array was performed every week over eight weeks, assuming that the pretreatment QA generally would suffice for all eight QA periods. In terms of potential clinical dose errors, the relative error for each week was adopted and added into the remainder of the 39 fractions as the actual dose-involved error. The mean ± SD dose differences for the three cases against the error-free dose grid for CTV and PTV, and also for the rectum and bladder, are shown in Figs. 4 and 5, respectively. The mean deviations and range (minimum and maximum values) between the first week and eighth week compared with the error-free dose grid and the actual dose-involved error are shown in Table 4. The values of the actual dose-involved error were close to those of the mean deviations between the first and eighth week. $D_{95\%}$ and the mean dose for the PTV, and $D_{98\%}$ and the mean dose for the CTV with the error-involved dose grid, were consistently negative in value compared with the error-free dose grid. $V_{40Gy}$ for the rectum, and $V_{65Gy}$ and $V_{40Gy}$ for the bladder were consistently positive. $V_{65Gy}$ for the rectum ranged from -10.75% to 13.66% with the error-involved dose grid. These ranges were relatively large, compared to those for other organs, because the irradiated volume was much smaller.

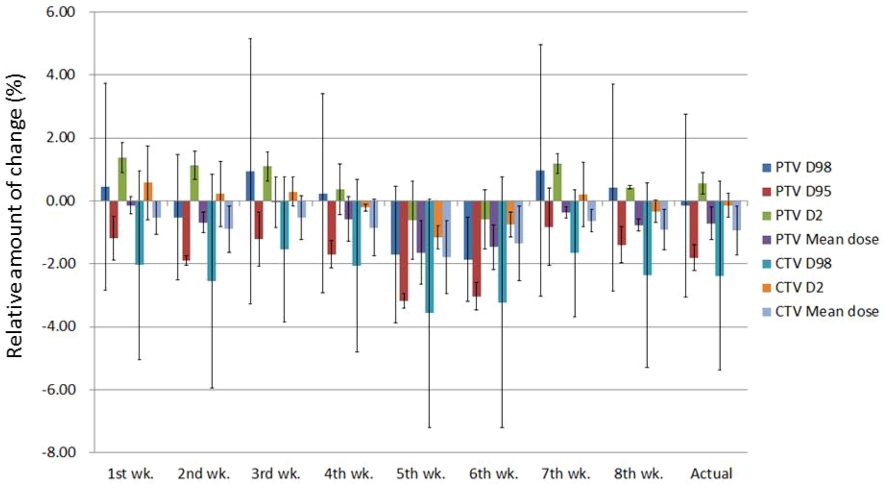

Fig. 4. Relative mean (bars: 1 SD) dose difference for three cases plotted against the error-free dose grid calculation for the CTV and PTV. The horizontal axis shows the time of measurement. The last column (Actual) shows the inclusion of the error measured in each week.

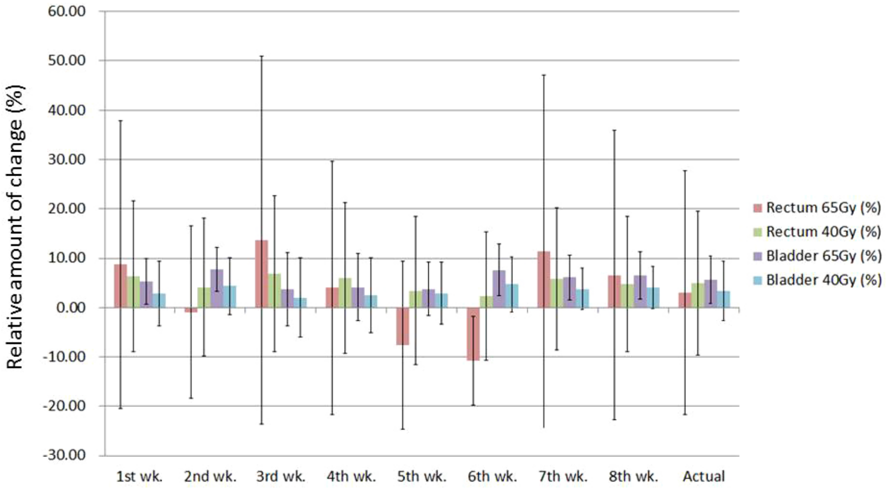

Fig. 5. Relative mean (bars: 1 SD) dose difference for three cases plotted against the error-free dose grid for rectum and bladder. The horizontal axis shows the time of measurement. The last column (Actual) shows the inclusion of the error measured in each week.

TABLE 4. Summary of the mean deviations and range, compared with the error-free dose grid calculations.

| *Volume Indices* | *Mean (range)*[a] (%) | *Actual*[b] (%) |
|---|---|---|
| PTV $D_{98\%}$ | -0.14 (-1.86–0.96) | -0.15 |
| PTV $D_{95\%}$ | -1.80 (-3.18– -0.82) | -1.81 |
| PTV $D_{2\%}$ | 0.55 (-0.62–1.38) | 0.55 |
| PTV mean dose | -0.71 (-1.64– -0.04) | -0.71 |
| CTV $D_{98\%}$ | -2.38 (-3.57–-1.55) | -2.38 |
| CTV $D_{2\%}$ | -0.14 (-1.16–0.57) | -0.14 |
| CTV mean dose | -0.94 (-1.79– -0.53) | -0.94 |
| Rectum $V_{65Gy}$ | 3.14 (-10.75–13.66) | 3.05 |
| Rectum $V_{40Gy}$ | 4.95 (2.30–6.79) | 4.96 |
| Bladder $V_{65Gy}$ | 5.62 (3.73–7.78) | 5.60 |
| Bladder $V_{40Gy}$ | 3.41 (2.04–4.72) | 3.40 |

[a] Mean deviations and range (minimum and maximum) are the results between the first and eighth week compared with the error-free dose grid calculations.

[b] Actual (%) denotes the results by the inclusion of the error measured each week compared with the error-free dose grid calculations.

## D. Dose changes due to MLC leaf calibration

MLC leaf calibration was performed once every two weeks, three times in total over eight weeks. The relative amount of change in volume indices was evaluated before and after each calibration. Figure 6 shows the mean relative amount of change (and standard deviation) for Cases 1, 2, and 3 at the time of each calibration. The mean relative amount of change was the relative difference before and after MLC leaf calibration. Most parameters, except $V_{65Gy}$ of the rectum, were less than 3%. $V_{65Gy}$ of the rectum was over 4%, and as much as 22% higher at the time of the third MLC leaf calibration. Again, the greater degree of variation in $V_{65Gy}$ for the rectum compared to those for the other parameters can be explained by the relatively smaller volume.

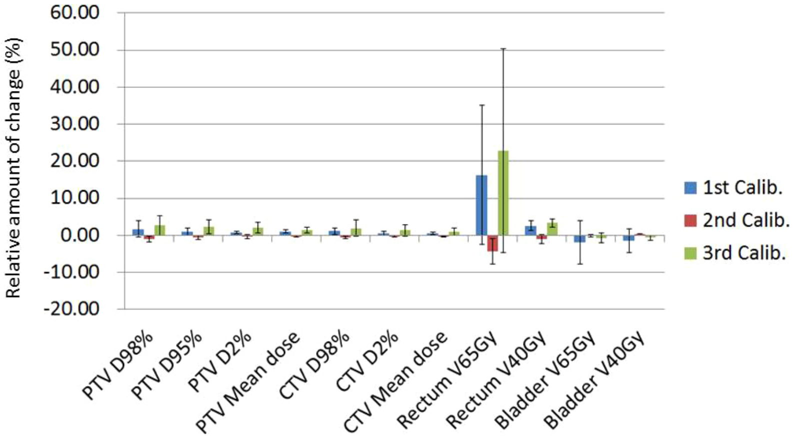

FIG. 6. Dose effects for the volume indices for each organ by MLC leaf calibration. Bars represent 1 SD.

## E. Comparisons in volume indices for the 20 cases

Table 5 summarizes the differences in volume indices compared with the error-free dose grid for the 20 cases in the first week. Among these 20 cases, the volume indices of $D_{98\%}$ and $D_{2\%}$ to the PTV, and the $D_{98\%}$ and $D_{2\%}$ to the CTV with error-involved dose grids were significantly

TABLE 5. Comparison of the error-free dose grid and error-involved dose grids for 20 cases.

| *Volume Indices* | *Error-free Dose Grid Mean (SD) (Gy)* | *Error-involved Dose Grid Mean (SD) (Gy)* | *p-value* |
|---|---|---|---|
| PTV $D_{98\%}$ | 63.52 (6.75) | 65.84 (5.11) | < 0.01 |
| PTV $D_{95\%}$ | 70.71 (3.88) | 70.82 (2.32) | Not significant |
| PTV $D_{2\%}$ | 81.58 (1.10) | 83.17 (2.06) | < 0.01 |
| PTV mean dose | 78.00 (0.19) | 78.51 (1.58) | Not significant |
| CTV $D_{98\%}$ | 76.17 (0.86) | 74.02 (2.14) | < 0.01 |
| CTV $D_{2\%}$ | 81.00 (1.00) | 82.27 (2.29) | < 0.01 |
| CTV mean dose | 78.82 (0.40) | 79.09 (1.90) | Not significant |
| *Volume Indices* | *Mean (SD) (%)* | *Mean (SD) (%)* | *p-value* |
| Rectum $V_{65Gy}$ | 4.42 (1.11) | 5.95 (1.81) | < 0.01 |
| Rectum $V_{40Gy}$ | 19.10 (1.79) | 21.27 (3.45) | < 0.01 |
| Bladder $V_{65Gy}$ | 21.45 (6.80) | 23.17 (8.60) | < 0.01 |
| Bladder $V_{40Gy}$ | 37.16 (12.28) | 39.58 (14.07) | < 0.01 |

different from those indices with the error free-dose grids. $V_{65Gy}$ and $V_{40Gy}$ for the bladder and the rectum were also significantly different.

## IV. DISCUSSION

Before the start of IMRT treatment, patient-specific QA is performed, such as absolute dose measurement with an ionization chamber, and dose distribution analysis with film or a diode detector. The QA results confirm whether settings are within the tolerance limits defined in our department. The phantom used in these measurements usually consists of a water-equivalent material, and is only a rough approximation of a human abdomen. If the QA results are within the tolerance limits from a physical point of view (such as the passing rate using 3%/3 mm criteria), it is difficult to determine whether the results affect the dose distribution inside the patient. Nelms et al.[5] concluded that there is a lack of correlation between conventional IMRT QA performance metrics (gamma passing rates) and dose errors in anatomic regions of interest.

We adapted the relative dose errors of each beam measured with beam-by-beam analysis using 2D diode detectors to the 3D dose grid data from the treatment planning system in DICOM RT format. A recently developed commercial software application (3DVH; Sun Nuclear Corporation, Melbourne FL) incorporates the beam-by-beam phantom dose back into the patient's images, structures, and treatment planning system dose using a "planned dose perturbation" (PDP) algorithm[7] to estimate the delivered patient dose and dose-volume histograms (DVHs) in three dimensions. The use of this software in dose evaluation has been described.[8,9] Using our own algorithm, we have developed in-house software to incorporate the 2D relative dose error into the 3D treatment planning dose calculation. To validate the modified method using the in-house software, the absorbed dose and the dose distributions were measured using the I'mRT phantom. The predicted doses to five measurement points compared with the measured absorbed doses were less than 2%, and mostly around 1% (Table 2). The predicted dose distributions showed good agreement with the film dose distributions shown in Fig. 3 ((f), (g), (h)). In order to predict the dose distribution with the error map in the patient CT images, the per-beam QA should be performed with a homogeneous phantom to create absorbed dose errors, and not fluence errors. Since the dose in the inhomogeneous patient CT images has already been calculated by the TPS, the relative dose differences for each beam can be incorporated to create a 3D dose distribution using the proposed modified method in this study.

For the three cases (1, 2, and 3) in Table 3, most volume indices, except the $V_{65Gy}$ to the bladder, were significantly different between the error-involved dose grids and the error-free dose grids. Yin et al.[15] noted that field size affects the detector response of the 2D diode detector array (i.e., when the diode array is calibrated with a 10 × 10 cm field, the dose it measures for smaller fields is lower and for larger fields is higher than an ionization chamber would measure). Olch[9] has shown the same evidence of a systematic 1% lower dose with the 3DVH system compared to TPS calculation. The equivalent size for all cases was smaller than 5 cm. When the dose output of a 5 × 5 cm field was compared with that of a 10 × 10 cm field using the 2D diode detector array, a 1.4% lower relative dose response was found. However, there was not the same tendency towards underdosing of around 1% in the error-involved doses, though the dose differences were both positive and negative.

In our previous study, we found that the MLC leaf positions gradually changed by as much as 1 mm over one month.[3] We proposed that this phenomenon could result in potential dose errors at the MLC leaf abutment regions, which create the dose distributions in the IMRT technique and, accordingly, performed MLC leaf calibration every two weeks to ensure the stability of distributions. MLC leaf calibrations were performed three times over eight weeks. Figure 6 shows the relative dose change before and after MLC leaf calibration. For the linear accelerator, the MLC leaf calibration is performed based on the light field edge, which should be fitted to graph paper at the nominal predefined positions of 20, 10, 0, and -10 cm in the X direction. Because the reproducibility of the procedure is operator-dependent, the same operator performed the MLC leaf calibration to ensure stability. Except for $V_{65Gy}$ of the rectum, the relative changes for the other volume indices were around 3%, indicating that the calibration procedure had good reproducibility.

Figure 7 shows an example of the dosimetric changes for the volume indices for each organ of one of the three cases. Although the dosimetric changes were small, the mean dose for the CTV and PTV, the $V_{65Gy}$ for the rectum, and the $V_{65Gy}$ for the bladder became gradually smaller in the three measurements before MLC leaf calibration. The decreasing dose to the bladder was of lesser magnitude than the dose to the other organs. Through a weekly MLC QA using the Picket Fence test, we confirm the pixel intensity between MLC leaf abutment regions. In the case that the intensity of an abutment region could change and lead to a reduction in dose, the MLC leaf calibration was eventually performed at its usual two-week interval, resulting in upward dose adjustment after the MLC leaf calibration. However, the procedure of leaf calibration is operator-dependent, so the dose adjustment may vary.

In Table 4 the actual dose differences, including weekly dose verification errors, were close to the average dose difference for all volume indices. In clinical radiation treatment, dose verifications, such as isodose verification with the 2D diode detector array and film, and absolute dose verification are routinely performed only once before treatment. In this study, we assessed the results of weekly dose verification over the whole treatment course of eight weeks. Results showed each fraction was not consistently affected by the same dose errors. This lack of consistency is because the reproducibility of the MLC leaf stop position is not stable, and the MLC leaf positions gradually change over the two weeks between calibrations. The dose errors have been averaged and reduced for total fractions.

The frequency of MLC leaf calibration varies from institution to institution. We recommend that the medical physicist should check and confirm how the MLC leaf position changes over at least one month, and then use that information to set calibration frequency.

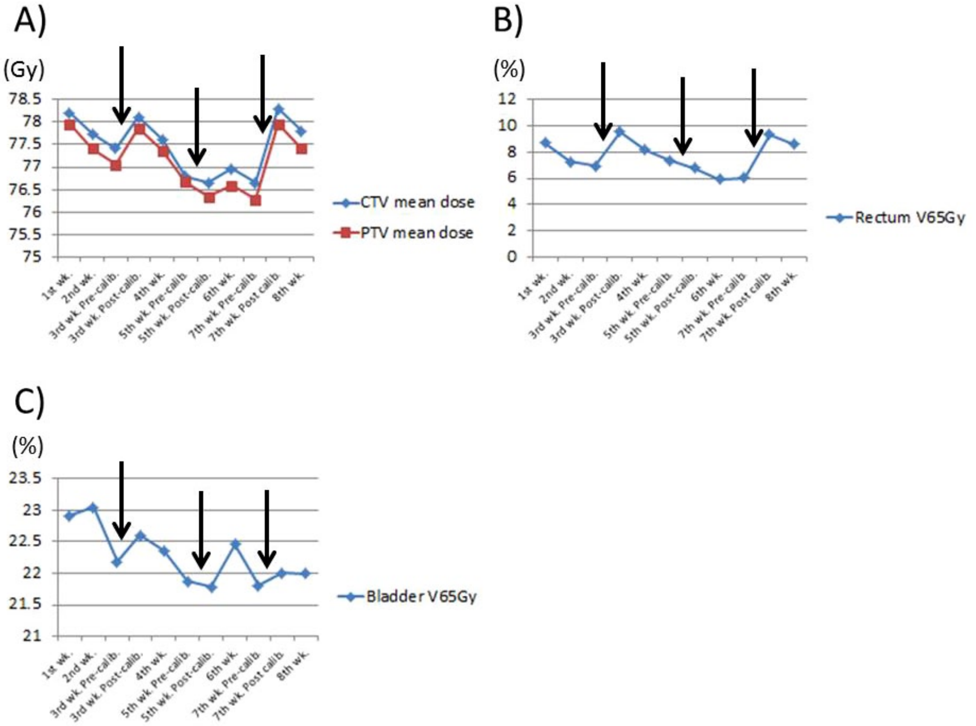

FIG. 7. Dosimetric changes for the volume indices for each organ: (a) the mean dose for the CTV and PTV; (b) $V_{65Gy}$ for the rectum; (c) $V_{65Gy}$ for the bladder. The horizontal axis shows the time of measurement. The black arrows indicate the MLC leaf calibration. Two measurements (precalibration and postcalibration for MLC leaves) were performed on the same day. Pre-calib. = Pre-MLC leaf calibration; Post-calib. = Post-MLC leaf calibration.

## V. CONCLUSIONS

Using beam-by-beam analysis, the dose measured with a 2D diode detector array at a 0° gantry angle was compared with the calculated dose, and the relative dose errors were factored into the actual 3D treatment planning dose grids using our in-house software. This proposed method modification was validated by the measurements of absorbed dose and dose distributions. Three prostate IMRT cases out of a total of 20 cases were used to test the measurement reproducibility, the dose error impact in the actual treatment plan for the volume indices of the target and normal tissues, and the dose effect from MLC leaf calibration. The dose error impacts created by the weekly measurements varied, and the total dose error impact was averaged over the whole treatment course of eight weeks. The dose error was insignificant for clinical dose evaluation. Even though the passing rate of the physical dose evaluation with the diode detectors was within tolerance, it is not stringent enough for dose evaluation in three dimensions. This predicted-dose approach is, therefore, useful for both medical physicists and clinicians.

## Supporting information

Supplementary Material

Supplementary Material
