# Supplementary figures and images for: Three‐dimensional dose prediction based on two‐dimensional verification measurements for IMRT
Source: J Appl Clin Med Phys. 2014 Sep 8;15(5):133–46. doi: 10.1120/jacmp.v15i5.4874 (PMC5711089; doi:10.1120/jacmp.v15i5.4874)

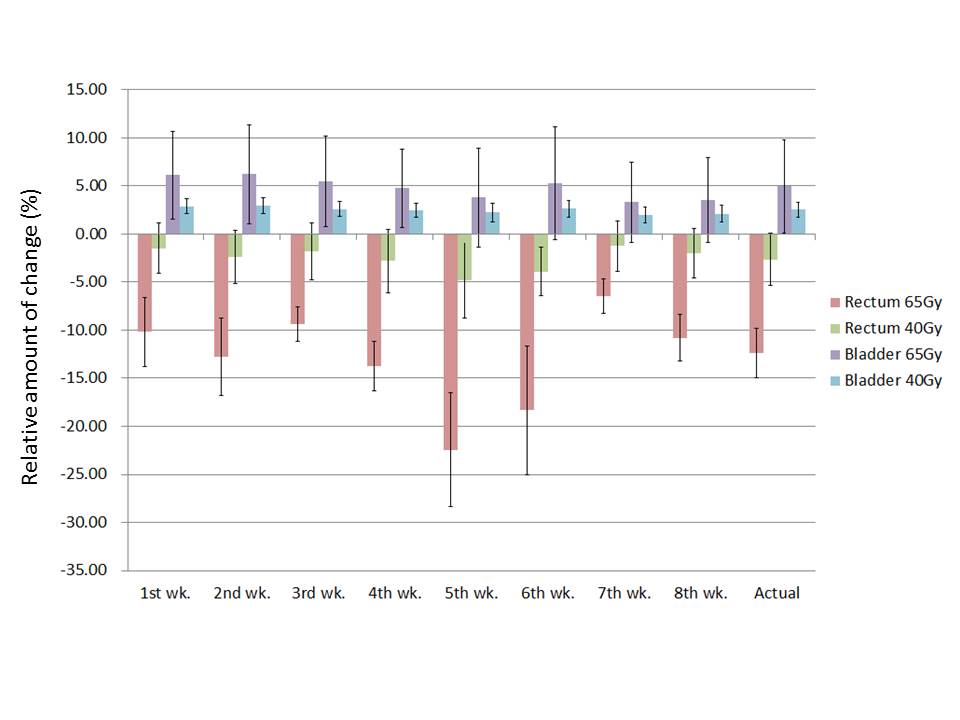

Supplement: Supplementary file 1 — Supplementary Material [file ACM2-15-133-s001.JPG]

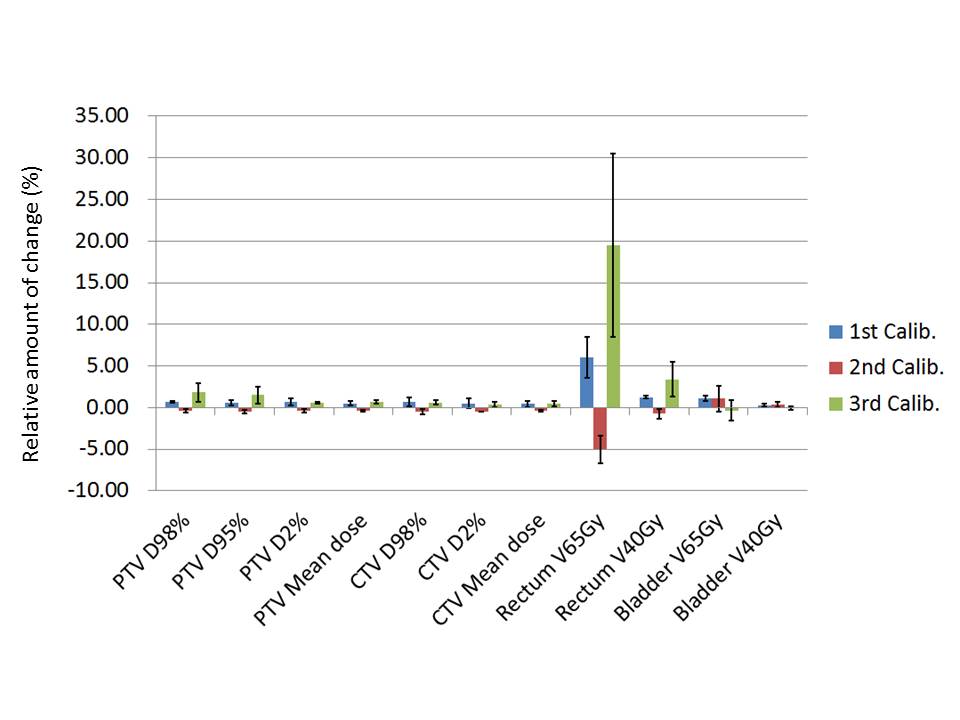

Supplement: Supplementary file 2 — Supplementary Material [file ACM2-15-133-s002.JPG]
